# Applying the theory of planned behavior to investigate type 2 diabetes patients' intention to receive injection therapy

**DOI:** 10.3389/fpubh.2023.1066633

**Published:** 2023-02-16

**Authors:** Su-Han Hsu, Kung-Pei Tang, Chia-Hui Lin, Pei-Chun Chen, Li-Hsuan Wang

**Affiliations:** ^1^Department of Pharmacy, Taipei City Hospital Yangming Branch, Taipei, Taiwan; ^2^School of Pharmacy, College of Pharmacy, Taipei Medical University, Taipei, Taiwan; ^3^Department of Pharmacy and Master Program, Tajen University, Pingtung City, Taiwan; ^4^Department of Education and Humanities in Medicine, School of Medicine, Taipei Medical University, Taipei, Taiwan; ^5^Department of Early Childhood and Family Education, College of Education, National Taipei University of Education, Taipei, Taiwan; ^6^Department of Pharmacy, Taipei Medical University Hospital, Taipei, Taiwan

**Keywords:** type 2 diabetes, shared decision making, patient decision aid, the theory of planned behavior, injection therapy

## Abstract

**Objectives:**

This study applied the theory of planned behavior (TPB) in shared decision making (SDM) to understand behavioral intention in patients with type 2 diabetes with regard to injection therapy for blood sugar control.

**Methods:**

A cross sectional study was conducted. Two hundred and fifty-four patients with type 2 diabetes participated this study and were interviewed by pharmacists in different clinics. A patient decision aid (PDA) entitled “Should I receive injection therapy regarding my type 2 diabetes condition?” was developed for this study and served as interview agenda which comprised 18 items to inquire their willingness to use injection therapy and related considerations during the SDM process.

**Results:**

The questionnaires were revised using item analysis, exploratory factor analysis, and a criteria of Cronbach's α > 0.7. This resulted in three constructs for all questionnaires that fit the TPB model. Attitude (β = 0.432; *P* < 0.001) and PBC (β = 0.258; *P* < 0.001) were directly correlated with intention. TPB explained 35.2% of the variance in intention toward the use of injection therapy.

**Conclusions:**

Attitude and PBC toward injection therapy positively and significantly influence the patients' intention to use injection therapy.

**Practical implications:**

These findings identify a key association for understanding behavioral intention in patients with type 2 diabetes with regard to blood sugar control during SDM.

## 1. Introduction

According to the International Diabetes Federation (1), individuals with diabetes aged 20–79 accounted for 9.3% of the total population worldwide in 2019. It is expected that the population of individuals with diabetes will increase to 10.9% by 2045. Although the global medical expenditure on diabetes exceeded US$760 billion in 2019, 4.2 million people worldwide died of diabetes or diabetes-related complications in the same year (1). The population with diabetes in Taiwan has been increasing steadily, from 830,000 in 2000 to 2.14 million in 2014, posing a public health challenge (2).

Stratton et al. found that for every 1% decrease in glycated hemoglobin (A1C) in patients with type 2 diabetes, the risk of myocardial infarction decreased by 14%, the risk of diabetes-related death decreased by 21%, and the risk of peripheral vascular disease decreased by 37% (3). The glucose-lowering medications for type 2 diabetes management comprise 2 dosage forms: (1) oral form, such as metformin for all patients; (2) injection form, such as insulin for patients who need high efficacy to achieve treatment goal, or the glucagon-like peptide-1 receptor agonists (GLP-1 RAs) for patients with high atherosclerotic cardiovascular disease risk (4, 5). DU (2) found that the prescription sharing of insulin increased from 7.78% in 2000 to 12.42% in 2014, while the GLP-1 RAs increased from 0.01% in 2011 to 0.19% in 2014 in Taiwan. The proportion of patients with diabetes using injection therapy among all patients with diabetes in Taiwan was much lower than that 20–30% in European and American countries, and this may affect overall diabetes control ratios.

In addition to pharmaceutical therapy, health education intervention has been widely used for blood sugar control in patients with diabetes (6, 7). For instance, Choi et al. found that health education intervention had significant effects on A1C control. According to the American Diabetes Association, the implementation of diabetes self-management education and support (DSMES) for patients with diabetes reduced A1C by ~1% (8, 9). DSMES focuses on personalization and patient-centered services, providing diabetes education, supporting patients in making their own decisions, and enhancing patients' self-care and health literacy. This is also the purpose of shared decision making (SDM) (10) and patient decision aids (PDAs) (11). For example, a meta-analysis by Karagiannis et al. indicated that PDAs had a positive effect on patient empowerment and engagement in the health care decision-making process (12).

Ajzen's Theory of Planned Behavior (TPB) (13) is useful for studying people's health-related behavior (14–16) and also diabetes patients' behavior (17, 18). According to the TPB, individuals' attitudes, subjective norms (SN), and perceived behavioral control (PBC) predict their behavioral intentions and actual performances. The term “subjective norm” refers to people's perceptions that are generally expected by significant others and the term “perceived behavioral control” represents an individual's beliefs about his/her ability to control over the behavior (13). The TPB has been incorporated in SDM (19, 20).

This study aims at investigating factors influencing type 2 diabetes patients' intention to use the injection therapy systematically. The theory of planned behavior is therefore adopted, which ascribed all influencing factors of human behavioral intention in three categories: attitudes, subjective norms and perceived behavioral control.

## 2. Materials and methods

### 2.1. Subjects and procedures

A cross sectional study with purposive sampling was conducted in the spring of 2021. Patients were included if they had type 2 diabetes; were aged 20 years or older; were taking oral hypoglycemic agents (OHAs); and were not using injection therapies, such as insulin or GLP-1 RAs. Verbal informed consent was received prior to participation. Two hundred and fifty-four patients with type 2 diabetes participated this study and have been interviewed by pharmacists in different clinics. A patient decision aid (PDA) entitled “Should I receive injection therapy regarding my type 2 diabetes condition?” is developed for this study and served as interview agenda, which comprised 18 items to inquire about their willingness to use injection therapy and related considerations during the SDM process. The study protocols were reviewed and approved by the Institutional Review Board at Taipei Medical University (TMU-JIRB No.: N202101079).

### 2.2. Measurement tool

The questionnaire for this study was developed as a PDA for SDM and was developed according to the guidelines of the Joint Commission of Taiwan (21, 22). The PDA comprised two parts: (1) a leaflet illustrating the pros and cons of OHAs and injection therapies with regard to outcome, adverse effects, and ease of use and (2) a questionnaire to identify the personal values of patients with type 2 diabetes and their attitudes toward injection therapies. Items in this questionnaire were based on the theory of planned behavior (TPB), which is a theory that links beliefs to behavior. According to TPB, an individual's behavioral intentions are shaped by three components: their attitude toward the behavior; subjective norms (SN), which is their belief that other people would approve of the behavior; and perceived behavioral control (PBC), which is their perception of their ability to perform the behavior (13).

A 5-point Likert scale (1 = *strongly disagree* and 5 = *strongly agree*) was used to measure a patient's intention to use injection therapy.

The questionnaire also asked questions related to demographic variables (gender, age, education level, year of diabetes mellitus diagnosis, BMI, number of OHAs currently being taken, blood sugar control status). An initial draft of the questionnaire was developed after a review of the literature on TPB (23–26). The first version included 25 items in four sections: (1) attitudes toward the use of injection therapy for diabetes (10 items); (2) SN toward using injection therapy for blood sugar management (7 items); (3) PBC with regard to injection therapy (6 items); and (4) intention to use injection therapy (2 items). A panel of nine experts was formed to examine the questionnaire's validity. Seven items with a content validity index <0.7 were eliminated. A focus group of 10 patients with diabetes was formed to review the modified questionnaire. The final version of the questionnaire had 18 items.

### 2.3. Statistical methods

#### 2.3.1. Item analysis and exploratory factor analysis

Item analysis was conducted to examine the validity and reliability of the questionnaire; the critical ratio, corrected item-total correlation, and Cronbach's alpha were calculated. An item was eliminated if its critical ratio was non-significant.

Factor analysis was conducted on items d1–d15 that had a satisfactory Kaiser–Meyer–Olkin (KMO) indicator of sampling adequacy (27). Subsequently, a principal component analysis (PCA) was conducted; items with eigenvalues >1 were included. Exploratory factor analysis using PCA and varimax rotation was conducted to determine the factor structure among items in the final study. An item was eliminated if its cross-factor loading was >0.4 for two or more components.

#### 2.3.2. Data analysis

The descriptive statistics of categorical variables, such as gender, education level, and year of diabetes diagnosis, are expressed as the frequency and percentage for each category. Continuous variables such as age, BMI, number of OHAs currently being taken, and blood sugar control status (A1C%, fasting blood sugar, abbreviate as glucose AC) are expressed in terms of the mean ± standard deviation (SD). Statistical analysis was performed using PASW Statistics Version 19.0 (SPSS, Chicago, IL, USA).

#### 2.3.3. Path analysis

**Figure 1:**
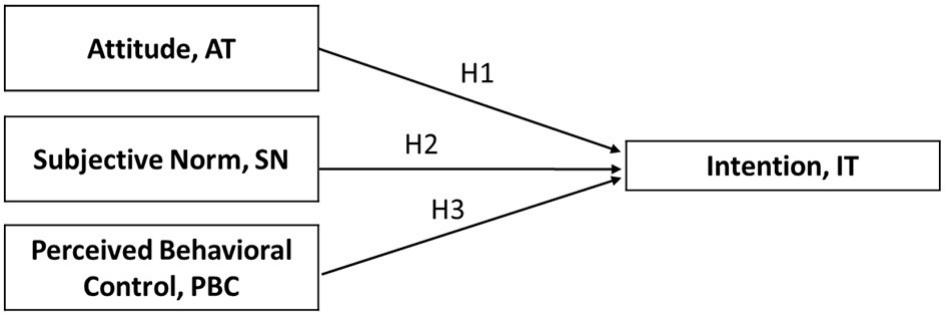
Conceptual model on the correlations of attitude, subjective norms, and perceived behavioral control with intention to receive injection therapy.

A path analysis was conducted using variables observed in structural equation modeling (SEM). SEM is used to uncover causal relationships (28, 29). A conceptual model is presented in Figure 1. Attitude, SN, and PBC were considered independent variables because they were expected to exert a direct effect on intention to receive injection therapy. Causal paths were depicted using arrows for this conceptual model. Statistical analysis was performed using IBM SPSS AMOS Version 22.0 (SPSS, Chicago, IL, USA).

The TPB-based following hypotheses were formulated (13):

H1: Attitude would have a positive and significant effect on intention in the use of injection therapy in patients with type 2 diabetes.
H2: Subjective norms would have a positive and significant effect on intention in the use of injection therapy in patients with type 2 diabetes.
H3: Perceived behavioral control would have a positive significant effect on intention in the use of injection therapy in patients with type 2 diabetes.

## 3. Results

### 3.1. Descriptive information

The descriptive statistics are shown in Table 1. Among the 254 patients, 249 participants were recruited (response rate: 98.03%). The mean age was 45.65 years (SD = 12.76 years), the mean BMI was 25.81, and 51.4% of the participants were male. More than half of the participants had senior high school or university as their highest education level. Almost 40% of the participants had DM for more than 10 years. The average A1C of participants was 7.44% (SD = 1.43%) and the average glucose AC was 143.64 mg/dL; 8.04 mmol/L (SD = 48.19 mg/dL; 2.70 mmol/L). The average number of OHAs taken was 2.11.

**Table 1:** Descriptive information of respondents' demographics (*N* = 249).

**Variable**	***N***	**%**	**Mean**	**SD**
Age			45.65	12.758
**Gender**				
Male	128	51.4		
Female	121	48.6		
**Education level**				
None	7	2.8		
Elementary	45	18.1		
Junior high	32	12.9		
Senior high	66	26.5		
University	99	39.8		
**Time since diabetes diagnosis** ^a^				
Less than a year	28	11.2		
1–5 years	61	24.5		
5–10 years	61	24.5	
More than 10 years	98	39.4		
A1C (%)			7.44	1.430
Glucose AC (mg/dL)			143.64	48.188
Glucose AC (mmol/L)			8.04	2.70
Numbers of concurrent oral hypoglycemic agents			2.11	1.008
BMI			25.81	4.79

a Totals may not add up to 249 due to incomplete demographic data.

### 3.2. Results of item analysis

Table 2 presents the results of the item analysis. The value for Cronbach's alpha was 0.864. All 15 items in the attitude, SN, and PBC sections of the questionnaire had a corrected item-total correlation >0.3 and were reserved for PCA. Item f1, “I would like to continue using OHAs,” had a corrected item-total correlation of < 0.3 (0.271) but was reserved because its critical ratio was significant (*t* = 3.541). Item f3, “I wish to discuss with my doctor, family, and friends before making a decision,” had a corrected item-total correlation of < 0.3 (*t* = −0.116) and was eliminated to increase the Cronbach's alpha from 0.864 to 0.883.

**Table 2:** Item analysis for the investigated items of scale based on the theory of planned behavior.

**Construct**	**Item number**	**Test for normality**			**Differentiation**	**Congeniality**		**Action**
		**Mean**	**Variance**	**Skewness**	**Critical ratio**	**Item-total correlation**	**Cronbach's** α **if item eliminated**	
Attitude	d1	3.48	2.151	−0.411	7.733^***^	0.521	0.855	Reserved
	d2	3.64	2.152	−0.644	7.488^***^	0.531	0.854^a^	Reserved
	d3	3.17	2.512	−0.114	11.896^***^	0.669	0.847^a^	Reserved
	d4	3.04	1.998	0.37	7.764^***^	0.511	0.855^a^	Reserved
	d5	3.43	0.934	−0.72	3.938^***^	0.333	0.862^a^	Reserved
	d6	3.37	2.316	−0.0.318	7.774^***^	0.448	0.859^a^	Reserved
Subjective norms	d7	3.63	1.205	−0.292	6.203^***^	0.517	0.856^b^	Reserved
	d8	3.80	1.010	−0.439	5.895^***^	0.501	0.857^b^	Reserved
	d9	3.15	1.826	−0.078	7.541^***^	0.513	0.855^b^	Reserved
	d10	3.20	1.798	−0.221	7.428^***^	0.502	0.856^b^	Reserved
Perceived behavioral control	d11	3.31	1.974	−0.170	11.082^***^	0.693	0.847^c^	Reserved
	d12	3.55	1.119	−0.265	6.540^***^	0.562	0.854^c^	Reserved
	d13	3.67	1.395	−0.642	6.957^***^	0.530	0.855^c^	Reserved
	d14	4.47	0.696	−1.288	5.484^***^	0.461	0.859^c^	Reserved
	d15	4.02	1.131	−0.709	8.918^***^	0.612	0.853^c^	Reserved
Intention	f1	2.36	2.011	0.638	3.541^**^	0.271	0.866^d^	Reserved
	f2	2.79	2.127	0.155	9.071^***^	0.662	0.848^d^	Reserved
	f3	2.86	2.101	0.026	−1.107	−0.116	0.883^d^	Eliminated
Cronbach's α							0.864	

a Construct for attitude, Cronbach's α = 0.711.

b Construct for societal norm, Cronbach's α = 0.761.

c Construct for perceived behavioral control, Cronbach's α = 0.790.

d Construct for intention, Cronbach's α = 0.756.

** P < 0.01,

*** P < 0.001.

### 3.3. Results of exploratory factor analysis

Following item analysis, PCA with varimax rotation was conducted. Table 3 presents the factor loading for each item above 0.4. An analysis of the 15 items revealed that 3 factors satisfied the KMO criterion with eigenvalues above 1, and the cumulative percentage of variance accounted for was 58.69%. KMO = 0.871 and Bartlett sphericity = 0.000, indicating that the three factors were suitable for factor analysis (27). The eigenvalues of the three factors were 6.287, 1.404, and 1.113 after varimax rotation (eigenvalues > 1). The three factors were consistent with the number of constructs expected in the research framework. After varimax rotation, the values of Cronbach's alpha for the three factors were 0.711, 0.761, and 0.790, and the three factors accounted for 41.91, 9.36, and 7.418% of the variance. These three factors refered to the constructs of TPB as (A) PBC, (B) attitude, and (C) SN. Five items (item d1, d5, d7, d8, d11) were either eliminated because their cross-factor loadings were >0.4 in 2 or more components, or they were moved to other constructs.

**Table 3:** Factor loading for the contributing items in questionnaire (*N* = 249).

**Construct**	**Item**	**Initial factors**			
		**Factor A**	**Factor B**	**Factor C**	**Communality**
Attitude	d1		0.483	0.570	0.559
	d2		0.793		0.684
	d3		0.811		0.749
	d4		0.432		0.389
	d5	0.629			0.461
	d6		0.551	0.405	0.481
Subjective norms	d7	0.484			0.415
	d8	0.727			0.529
	d9			0.896	0.895
	d10			0.894	0.894
Perceived behavioral control	d11	0.438	0.665		0.673
	d12	0.711			0.641
	d13	0.572			0.431
	d14	0.656			0.515
	d15	0.535	0.430		0.486
Eigenvalues		6.287	1.404	1.113	
% of variance		41.91%	9.36%	7.418%	
Cumulative %		41.91%	51.27%	58.69%	
Kaiser–Meyer–Olkin measure of sampling adequacy (KMO)		0.871			
Bartlett's test of sphericity		~χ^2^ = 1834.688^***^			

*** P < 0.001.

### 3.4. Results of path analysis

In the first step of the analysis, correlations between these constructs were examined for the scores in attitude, SN, PBC, and intention. Then, a path analysis was conducted based on SEM. The correlation values obtained from the analyses are presented in Table 4.

**Table 4:** Correlations among variables.

**Variables**	**1**	**2**	**3**	**4**
1. Attitude	1.000			
2. Subjective norms	0.507^***^	1.000		
3. Perceived behavioral control	0.594^***^	0.478^***^	1.000	
4. Intention	0.467^***^	0.200^***^	0.426^***^	1.000

*** P < 0.001.

As seen in Table 4, all variables exhibited positive correlations. Attitude was positively correlated with intention (*r* = 0.467; *P* < 0.001), SN was positively correlated with intention (*r* = 0.200; *P* < 0.001), and PBC was positively correlated with intention (*r* = 0.462; *P* < 0.001).

**Figure 2:**
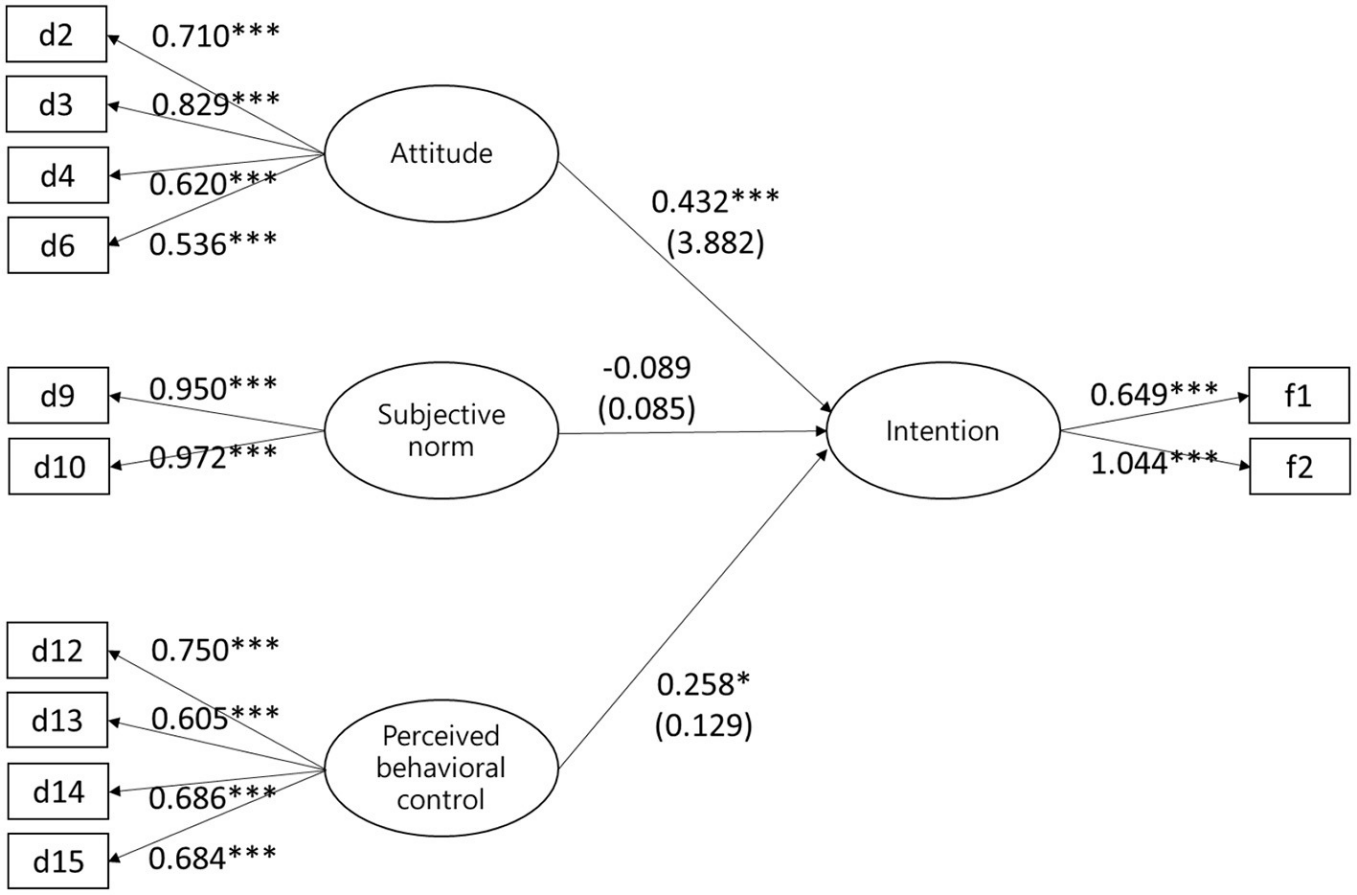
Path analysis of the model. *P < 0.05, ***P < 0.001.

The path diagram and standardized predicted values obtained from the analysis conducted in the conceptual model are presented in Figure 2. The model fit results were good (root mean square error of approximation = 0.053) or excellent (χ^2^/*df* = 1.699; CFI = 0.981; TLI = 0.973; GFI = 0.959; AGFI = 0.93; SRMR = 0.0558). Estimates for the model are presented in Table 5.

**Table 5:** Analysis of path analysis model.

**Path between the variables**			**Estimate (β)**	**S.E**.	**C.R.(t)**	***P***	**Variances**	**SMC**
Intention	←	Attitude	0.432	0.138	3.882	***	0.459	0.352
Intention	←	Subjective norm	−0.089	0.085	−1.303	0.193		
Intention	←	Perceived behavioral control	0.258	0.129	2.492	0.013		

*** P < 0.001.

As indicated in Table 5, some paths between the variables in the research model were significant (*P* < 0.05). Specifically, attitude (β = 0.432; *P* < 0.001) and PBC (β = 0.258; *P* < 0.001) had a direct positive effect on intention in the use of injection therapy, whereas the effect of SN was non-significant. According to Table 5, attitude, SN, and PBC explained 35.2% of the variance in intention in the use of injection therapy.

The values for total, direct, and indirect effect presented in Table 6 indicated that attitude, SN, and PBC had no indirect effect on intention. On the basis of these findings, H1 and H3 were accepted and H2 was rejected.

**Table 6:** Total, direct, and indirect effects of constructs on the intention toward the use of injection therapy.

**Independent variable**	**Dependent variable**	**Direct effect**	**Indirect effect**	**Total effect**	**Hypothesis**
Attitude	Intention	0.432^***^	–	0.432	Accepted
Subjective norms	Intention	−0.089	–	–	Rejected
Perceived behavioral control	Intention	0.258^*^	–	0.258	Accepted

* P < 0.05,

*** P < 0.001.

## 4. Discussion and conclusion

### 4.1. Discussion

#### 4.1.1. Comparison with existing studies

In total, 249 participants were recruited; the mean age was 45 years, the gender ratio was 105.8 males per 100 females, the mean A1C was 7.44%, and glucose AC was 143.64 mg/dL; 8.04 mmol/L. The demographic variables of the participants were compared with current epidemiological data on type 2 diabetes in Taiwan. The average of onset of type 2 diabetes in Taiwan is ~59.5 years (2). In other words, the participants in this study may on average be younger than patients with diabetes in the rest of Taiwan. Gender and glycemic control status were similar to those reported in other epidemiological studies in Taiwan (2, 30). In this study, 9.6% of participants used four or more OHAs. In 2014, the proportion of Taiwanese patients with diabetes using more than four types of drugs (OHAs and injection therapy) accounted for only 5.32% (2). A comparison of the two results implies that the patients in this study may need to use more OHAs because the patients are unwilling to use or want to delay the use of injection therapy. As a result, the number of OHAs being used increases, thus this study could use TPB to find out why patients are reluctant to use injection therapy.

In this study, attitude and PBC positively and significantly affected intention in the use of injection therapy for patients with type 2 diabetes. SN was not found to affect intention to use injection therapy.

With regard to attitude, patients with type 2 diabetes were more likely to use injection therapy if they felt that their life would not be changed by injection therapy, had no concerns regarding side effects, were not afraid of pain, and were willing to administer injections on themselves. One study in Singapore (31) found that fear of pain and fear of lifestyle disruption from injection therapy affected patients' willingness to use injection therapy. This finding was similar to our study if patients did not expect life-changing would have more positive attitude toward injection therapies. A study in China (32) found that fear of side effects affected patients' willingness to use injection therapy. Likewise, a study in Saudi Arabia (33) found that the fear of side effects and the fear of hypoglycemia affected patients' willingness to use injection therapy. Another study in South Africa (34) found that fear of injections and fear of needles made patients reluctant to use injection therapy. Two studies in the United States (35, 36) found that fear of pain affects patients' willingness to use injection therapy or GLP-1 RAs. A qualitative study reported a similar result that the pain of injection would be one of the factor (18). In conclusion, fear of the pain from injection therapy affects willingness to use injection therapy. If a patient has a negative attitude, including fear of needles, fear of pain, fear of side effects, or fear of drugs, medical professionals might be able to change the patient's attitude through health education (37). If a patient is afraid of pain or needles, explaining to the patient that the current needles are very thin and short may be helpful. Showing samples can improve a patient's attitude toward needles. If a patient is already self-monitoring their blood sugar, informing the patient that injection therapy is less painful may be helpful. These actions reduce the patient's negative attitude toward the use of injection therapy for diabetes.

With regard to PBC, a patient's intention to use injection therapy increases if they believe that A1C can be better controlled using injection therapy; if they believe that someone can help when they encounter difficulties during use; if they can afford the injection therapy; and if they can find suitably concealed places to use injection therapy. Many studies have shown that a patient's ability to use insulin affects their willingness to use insulin (18, 23, 31, 34). Improving the ease of use of injection needles is key to enhancing the patient's PBC. Hypoglycemic injector pen devices, in popular use today, eliminate the need to use a syringe to remove the medication from the vial with the use of an integrated syringe instead. They are convenient to use and easy to carry. In particular, some medicines are designed for fixed-dose application to simplify usage. In addition to the ability to use pen needles, as reported by studies in China, South Africa, and the United States, cost is a key factor influencing a patient's decision to use injection therapy (32, 34, 36). Although Taiwan's National Health Insurance program covers the majority of costs to patients whether they use OHAs or injection therapy, injection therapy is still cost more than OHAs because extra costs for pen needles and alcohol pads, which affects intention regarding the use of injection therapy.

SN was not shown to influence intention in the use of injection therapy. The significant others of participants selected in this study such as colleagues or close friends are not a main reason to influence on participants' intentions in using injection therapy may be a possible reason. A behavioral intentions study in Brazil found that the opinions of patients' children influenced patients' insulin use (18). Therefore, the significant others selected for this study colleagues or close friends may be less appropriate. In addition, many studies have found that SN is the weakest predictor of behavioral intention (38). In a study using TPB to investigate medication adherence in patients with chronic diseases, SN was the weakest predictor (39). In 2021, a randomized, controlled trial in Malaysia using the TPB to study medication compliance in patients with diabetes obtained similar results: SN were not significantly correlated with behavioral intentions (40). This study supported our findings that SN were not significantly correlated with intentions.

The TPB is appropriate for the study of voluntary behavior, especially health-related behavior (15). The TPB can be appropriately applied to investigate the behavioral intentions of patients in the use of injection therapy. In summary, attitude has the highest correlation with behavioral intention, PBC has the second highest correlation, and SN has no correlation. The results of this study are similar to those of other studies on TPB. One systematic review and meta-analysis of 13 studies of adolescent nutrition-related behavioral intentions found that attitude had the highest correlation with behavioral intentions (41). Another systematic review and meta-analysis of intentions for cervical screening found that attitude had the highest correlation with intentions (42). There is also a recent systematic review and meta-analysis regarding the use of sunscreen; that study that attitude had the highest correlation with behavioral intentions, followed by PBC and SN (43).

#### 4.1.2. Strengths and limitations of the study

The average age of the participants was lower than that of the overall population of patients with diabetes in Taiwan, which may imply that this study included a younger population of patients with diabetes. Behavioral intentions may differ between younger and older people with diabetes. This may impede generalizability. Furthermore, the scores of intentions in this study could not be directly converted to indicate whether injection therapy should be used, and it was also impossible to track whether A1C in patients with type 2 diabetes could be better controlled as a result. A long-term longitudinal study should be conducted to better reveal a connection between the decision result and the control of blood glucose, thus elucidating the substantive effect of the use of SDM interventions.

Furthermore, the use of the TPB alone may be insufficient to understand patient behavioral intentions. The TPB constructs also do not encompass emotion, which is a key part of the irrationality that people exhibit in real life (44). Every TPB study is a study of the behavioral intentions at a single point in time rather than over the long time. However, a person's behavioral intention changes over time.

In particular, the advantage of this study is its combination of TPB with SDM. Through performing SDM in clinical practice, one can understand a patient's attitude, SN, and PBC through PDA. Medical personnel will find such PDA useful. The sample size of this study was 249, which met the requirements of confirmatory factor analysis (45). The ratio of participants to estimated parameters should be more than 1:10, and, per the MLE method, which was used in this study, the sample size should be more than 200 (46). Only a few participants dropped out during the research process; all items were reliable and valid; and the Likert scale had no common ceiling effect. No missing values were present in the TPB section of the study, which indicates high study quality.

### 4.2. Conclusion

Despite that, the PDA has been used for enhancing the SDM process (11), there was not a common theoretical framework to inquiry diabetic patients' acceptance and considerations about injection therapy. The TPB is therefore adopted in this study and incorporated into our PDA for diabetes education. This study showed that attitude and PBC have positive and significant influence on patients' intention to use injection therapy. Patients with type 2 diabetes with positive attitudes had a stronger intention to use injection therapy than those with negative attitudes. Patients with higher positive PBC had a stronger intention to use injection therapy than those with low positive PBC. SN had no influence on intention.

### 4.3. Practical implications

This study developed a reliable and valid PDA. The TPB part of this study showed that attitude and PBC have a positive and significant influence on patients' intention to use injection therapy. Therefore, during SDM, when a patient exhibits a negative attitude, such as fear of needles, pain, or adverse effects, physicians should attempt to change the patient's attitude through education. With regard to the elimination of the obstacles related to PBC, teaching patients on the correct use of medication and improving patients' injection device skills might increase their confidence. We present a useful method for understanding the intention of patients with type 2 diabetes in blood sugar control during SDM.

## Data availability statement

The original contributions presented in the study are included in the article/supplementary material, further inquiries can be directed to the corresponding author.

## Ethics statement

The studies involving human participants were reviewed and approved by Taipei Medical University-Joint Institutional Review Board. Written informed consent for participation was not required for this study in accordance with the national legislation and the institutional requirements.

## Author contributions

S-HH and K-PT contributed to conception and design, analysis and interpretation of data, drafting the article and revising it critically for important intellectual content, and final approval of the version to be published. C-HL contributed to investigation, data curation, and interpretation of data. P-CC contributed to investigation and writing original draft preparation. L-HW contributed to conception and design, analysis and interpretation of data, drafting the article, revising it critically for important intellectual content, and final approval of the version to be published. All authors contributed to the article and approved the submitted version.
